# An AI-Driven Model to Enhance Sustainability for the Detection of Cyber Threats in IoT Environments

**DOI:** 10.3390/s24227179

**Published:** 2024-11-08

**Authors:** Majid H. Alsulami

**Affiliations:** Applied College, Shaqra University, Shaqra 11961, Saudi Arabia; malsulami@su.edu.sa

**Keywords:** cyber threats, cyber security, artificial intelligence (AI), Artificial Fish Swarm-driven Weight-normalized Adaboost (AF-WAdaBoost), IoT environment

## Abstract

In the face of constantly changing cyber threats, a variety of actions, tools, and regulations must be considered to safeguard information assets and guarantee the confidentiality, reliability, and availability of digital resources. The purpose of this research is to create an artificial intelligence (AI)-driven system to enhance sustainability for cyber threat detection in Internet of Things (IoT) environments. This study proposes a modern technique named Artificial Fish Swarm-driven Weight-normalized Adaboost (AF-WAdaBoost) for optimizing accuracy and sustainability in identifying attacks, thus contributing to heightening security in IoT environments. CICIDS2017, NSL-KDD, and UNSW-NB15 were used in this study. Min-max normalization is employed to pre-process the obtained raw information. The proposed model AF-WAdaBoost dynamically adjusts classifiers, enhancing accuracy and resilience against evolving threats. Python is used for model implementation. The effectiveness of the suggested AF-WAdaBoost model in identifying different kinds of cyber-threats in IoT systems is examined through evaluation metrics like accuracy (98.69%), F-measure (94.86%), and precision (95.72%). The experimental results unequivocally demonstrate that the recommended model performed better than other traditional approaches, showing essential enhancements in accuracy and strength, particularly in a dynamic environment. Integrating AI-driven detection balances offers sustainability in cybersecurity, ensuring the confidentiality, reliability, and availability of information assets, and also helps in optimizing the accuracy of systems.

## 1. Introduction

Information and communication technologies have evolved over the past century, resulting in massive changes to conventional network communication patterns. The Internet of Things (IoT) has emerged as an effective tool for network communication by introducing modern ideas of information transfer via networks, an emerging phenomenon in every area of life [1]. The IoT has grown to become a fundamental part of the modern world. Smart homes, smart automobiles, and a host of other innovations empower the global community. Additional advantages of the IoT include communication, automation, control, and monitoring [2]. However, the explosive adoption of IoT devices also poses an important security risk due to the lack of hardware security support and security measures. Conventional protection techniques are not viable on IoT devices due to their limited processing power. Nevertheless, while streamlining these techniques is an accepted fix, it may result in significant security vulnerabilities across the entire system [3].

As the number of IoT associations increase, unanticipated malicious attacks are becoming increasingly frequent. The objective of these malicious software (or malware) attacks is to breach the online security of mobile phones, PC frameworks, and IoT devices. In order to counter malware attacks, increasingly sophisticated methods of identifying malware are being developed. Malware detection is divided into two distinct approaches: static and dynamic procedures, both fundamentally differing in their approach to identifying malware [4]. However, maintaining adequate security for IoT devices is an ongoing challenge due to their limited processing power and communication capacity, which is responsible for the vulnerability of IoT devices to network layer attacks such as denial of service (DoS) [5]. Thus, in order to identify cyber threats in an IoT environment, this study recommends an innovative artificial intelligence (AI)-based approach. The security of IoT settings is improved through the implementation of a novel Artificial Fish Swarm-driven Weight-normalized Adaboost (AF-WAdaBoost) model, which enhances the accuracy and effectiveness of malicious attack detection.

Although IoT devices have undergone rapid development, cybersecurity remains an open problem, as traditional approaches are not always successful in dealing with new and previously unknown security threats [5,6]. Additionally, existing detection systems also have shortcomings in terms of their ability to differentiate between true and false positive and negatives, and tend to be slow in examining threats, which present weak points that hackers can exploit to access confidential data and disable services [7].

This research seeks to fulfill this gap in cyber security by recommending and implementing an AI-based system referred to as AF-WAdaBoost, intended to detect cyber threats in IoT networks, and which will be shown in the following sections to have high accuracy, precision, and system performance.

The researchers aim to enhance threat detection and mitigation in IoT environments using AI technology. The research aims to evaluate the model’s scalability, adaptability to threats, integration with existing cybersecurity infrastructure, and performance in the real world. The model has potential to enhance the security and resilience of IoT deployments, and influence regulations and standards for IoT environments in sectors such as smart homes, industrial automation, healthcare, and transportation.

The study emphasizes the urgent need for enhanced cybersecurity measures when it comes to IoT systems being targeted by a growing number of cyber threats. The proposed system utilizes Artificial Fish Swarm-driven Weight-normalized Adaboost (AF-WAdaBoost) to maximize accuracy and efficiency in identifying attacks. The proposed model was trained based on real-world IoT network traffic data under different attack scenarios. Using Python 3.10 software, it preprocessed the raw information using min-max normalization. These experimental results will help demonstrate that the suggested AF-WAdaBoost model can be used to identify different kinds of cyber threats in IoT systems. The findings will demonstrate essential improvements in accuracy and strength, especially in dynamic environments. This study provides a comprehensive background on the growing security risks posed by IoT devices, as well as the need for advanced cybersecurity solutions. An innovative AI-driven system is then presented, followed by experimental details and results that demonstrate its effectiveness in detecting cyber threats.

A summary of the contributions of the research is given below:

- The study presents and evaluates a novel AI-based cybersecurity model for IoT systems, combining the Artificial Fish Swarm Optimization with Weight-normalized Adaboost;
- The proposed model improves the accuracy and precision of threat classification;
- The AF-WAdaBoost model outperforms the state-of-the-art models, without increasing the resource strain on the system;
- The model has the potential to improve the sustainability of IoT systems by reducing the wastage of resources in identifying and handling threats.

## 2. Related Work

The Internet of Things, or IoT, has become a new technological platform redefining how devices can be interconnected and held in various sectors. IoT technologies refer to a complex system of connected devices that gather and share information to support automation and optimization across various industries, including healthcare, home automation, industrial applications, and transportation. In healthcare, IoT devices are used to monitor and manage the status and conditions of patients, and this brings about better results and lower expenses [8]. Smart homes make use of the IoT to promote enhanced security, efficient energy utilization, and physical comforts through connected home appliances and structures. Some of the use cases of the IoT in industries are predictive maintenance, asset tracking, and managing processes to enhance productivity and cut down on potential losses due to equipment failure. In the field of transportation, the IoT is useful in smart traffic control, managing fleets and self-driving cars, and improving transportation safety [9]. Based on the above applications, the IoT is indispensable in advancing society and enhancing the quality of life in multiple aspects.

While IoT technology has many benefits, these are offset by the security concerns resulting from the higher attack area and the variety of devices connected. Due to the constraints in the processing capability of many IoT devices, they are easily targeted and hard to protect. Some of the typical security issues that have been identified in IoT networks include insecure authentication processes, the lack of encryption, and most importantly, inadequate security patches as malicious individuals attempt to gain full control of IoT systems [10]. Due to the wide distribution of IoT devices, it becomes hard to implement sound security systems since most devices are designed to communicate with other heterogeneous devices and interact in intricate ways. In distributed denial of service (DDoS) attacks, perpetrators use the computational resources of compromised IoT devices to flood the targeted servers, a strategy which has been on the rise. Security and privacy are also an issue because IoT devices are used to gather large volumes of data about their users, which needs to be safeguarded from hacking or other breaches. These challenges draw attention to the necessity to develop proper cybersecurity solutions that will consider the peculiarities of IoT environments [11].

With the advancement in technology, artificial intelligence (AI) has been proven to play a significant role in reinforcing cybersecurity protections that can identify and address threats on their own in real time. Neural networks, decision trees, and support vector machines are popular machine learning techniques that can be employed to analyze patterns and outliers that may show signs of security threats. For example, IDSs that integrate AI technology can examine the flow of traffic or communication networks to detect the presence of destructive activities in organization environments and alert them of impendent attacks [12]. With AI, threat intelligence is another area that can also be automated, whereby huge amounts of threat data from different sources can be mined to discover new and emerging threats. In the context of the IoT, AI techniques can be most efficient in the case of tackling SSI since some of the AI approaches can be considered adaptive and scalable. Still, the constantly evolving nature of cyber threats requires updating and refining the AI models used, which may lead to a decrease in their effectiveness in the future [13].

The concept of sustainability in IoT networks and cybersecurity is significant to catalyze the attainment of sustainable development goals (SDGs). The use of IoT devices is rapidly increasing, and these systems must be protected from cyber threats which can interrupt other important infrastructures, causing negative environmental and economic consequences. AI-based solutions like the AF-WAdaBoost model not only improve the cybersecurity of these systems but also promote sustainability by providing better system flexibility and decreasing the need for replacing these systems frequently due to cyberattacks [14]. These improvements contribute to SDG 9 (Industry, Innovation, and Infrastructure) by encouraging resilient infrastructural development and sustainable industrialization [15]. Furthermore, protecting IoT systems can help minimize environmental impacts due to cyber incidences to correspond with climate action (SDG 13) on the cybersecurity level by lowering the effects that are usually incurred during the recovery stage [16]. Hence, incorporating advanced AI for IoT cybersecurity is crucial for long-term technological and environmental security [17].

This is highlighted in [18], which presents a theoretical discussion and research on the relationships between cybersecurity and sustainable development in inter-organizational networks. The research notes the importance of cybersecurity in green technological development, as the risk of exposing systems to cyber threats can make producers feel concerned about automating the processes involved in their activities. This can drag down the development of information communication technology (ICT), thus slowing down green development.

Ref. [19] carried out a systematic literature review exploring the critical intersection of cybersecurity measures and green technologies with a focus on assessing their combined impact on the sustainability goals and the implications to stakeholders. The study identified several key themes, including the integration challenges and opportunities of cybersecurity within sustainable technologies; the evolving landscape of cybersecurity protocols; and the strategic implications for industry leaders, policymakers, and technologists. The study found there was a dual imperative of pursuing sustainability alongside security, highlighting the importance of integrating robust cybersecurity measures while maintaining the environmental benefit of green technology. One challenge to achieving this goal that the study identified were the rapid evolution of cyber threats. This highlights the need for a dynamic system that can adapt to evolving cyber threats without the need for excessive oversight.

Ref. [20] notes that the cybersecurity risks to things, sensors, and monitoring systems in IoT sustainability are distinct from conventional networking systems. IoT networks have much stricter requirements in terms of energy efficiency, safety, power, and performance requirements when compared against conventional devices.

However, it is crucial to point out a few research limitations, particularly concerning the application of AI techniques in enhancing cybersecurity for IoT. One of the major drawbacks highlighted is the lack of large datasets to model the diverse and complex nature of IoT threats [9]. Most of the datasets available as of date cover a broad range of applications, are not updated in terms of time, and do not encompass generalizing and running an AI model in real-world situations. Furthermore, challenges are observed when implementing AI in IoT security systems since the number of calculations and their complexity are directly linked to IoT devices’ capabilities [10]. This concern is somewhat underrepresented in more recent works focusing on the establishment of AI-based IoT systems. However, there is still worry about the ability of the AI decision-making process because, due to deep learning, a security analyst cannot have confidence in AI-driven alerts. It is also important to recognize their stability against adversarial attacks where the attacker tries to produce desired inputs that the model fails to detect [12]. These are critical gaps in establishing state-of-the-art IoT cyber security strategies and the application of AI in securing IoT systems. While IoT technologies introduce opportunities, novelties, and advancement prospects to different industries, they also open vast cyber security threats that demand new approaches. Several studies illustrate the application of AI in enhancing cybersecurity; however, some issues in the existing literature should be discussed, including the insufficiency of big data, non-intrusive AI models, interpretability of AI, and semi-oriented attacks. These gaps can be bridged by the proposed AI-based system known as AF-WAdaBoost, which will improve the ability to detect cyber threats in IoT networks and, consequently, improve the security and sustainability of the given solution.

## 3. Materials and Methods

**Figure 1:**
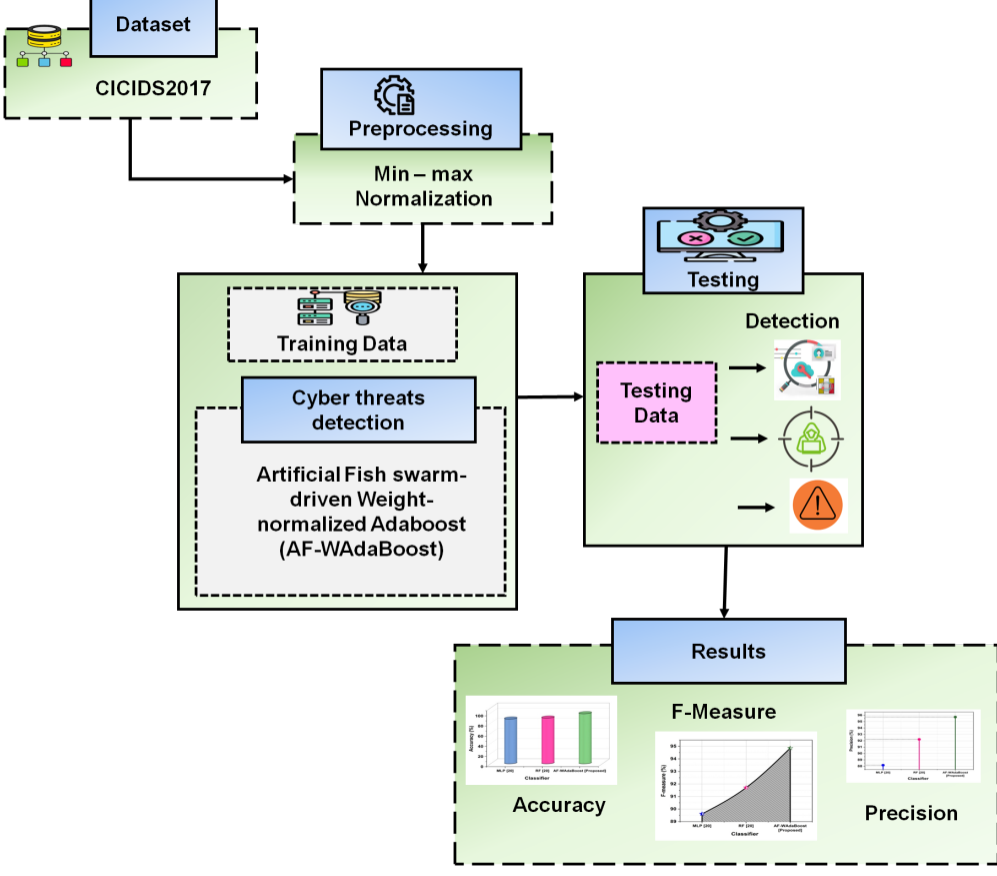
Flowchart for the proposed method.

This section presents an integrated approach for the recommended AF-WAdaBoost strategy of cyber threat detection in IoT settings, and a flowchart of the suggested method is illustrated in Figure 1.

### 3.1. Data Collection

In this research three datasets were obtained from Kaggle to conduct cyber threat detection: CICIDS2017 (https://www.kaggle.com/datasets/dhoogla/cicids2017, accessed on 10 April 2024), NSL-KDD (https://www.kaggle.com/datasets/hassan06/nslkdd, accessed on 10 April 2024), and UNSW-NB15 (https://www.kaggle.com/datasets/dhoogla/unswnb15, accessed on 10 April 2024). These datasets are utilized to train and assess the suggested AF-WAdaBoost model, which aims to improve the identification of various cyber threats in network environments.

### 3.2. Data Pre-Processing Using Min-Max Normalization

Certain features have higher values than others, which might cause a model to be biased in favor of the large characteristic values and lead to inaccurate results. Since normalization enables the features to be scaled within a range of [0.0, 1.0] without altering the normalcy of data behavior, it is crucial to prevent features with large values from outweighing features with lower values. Scaling feature values inside the range of [0.0, 1.0] is accomplished via the min-max normalization approach, as seen in Equation (1).

(1) $$W=\frac{\left(b-b_{min}\right)}{b_{max}-b_{min}}$$

where W is the normalized value, b is the original value, and b_max_ and b_min_ are the features of the minimum and maximum values, respectively.

### 3.3. Cyber Threat Detection Using Artificial Fish Swarm-Driven Weight-Normalized Adaboost (AF-WAdaBoost)

This section provides a brief explanation of the ML method that was employed in the cyber threat detection experiment, AF-WAdaBoost.

#### 3.3.1. Artificial Fish Swarm Optimization

The AF optimization method simulates attacker behavior, including foraging and motility, to identify attacks. For instance, the majority of fish in a pond will usually be found where the most food is available. Chasing, swarming, and preying are the three primary phases in the AF. These three processes are repeated in the AF to obtain the best response for cyber threat detection in IoT devices. The AF uses a meta-heuristic to continuously search for potential solutions to find the most satisfying solution in a finite amount of time, much like other bio-inspired algorithms. For the AF, each fish’s location is regarded as an attack solution, and each solution is assigned a fitness value according to the fitness function. Changing the goals leads to a shift in the fitness function of threat detection.

##### Route Selection Process

An optimal solution search issue is guaranteed by AF. Examining the state vector and a swarm of N imaginary fish, $\;Y=(y_{1},y_{2},\ldots y_{n})$, the AF process is used to improve the artificial fish n states/attributes. Furthermore, suppose that X=f(Y) represents the cyber threat detection function that gives the artificial fish food concentration at its current location and that $E_{ij}=\left\|y_{i}-y_{j}\right\|$ is used to characterize the separation between artificial fish j and i. The greatest number of steps the fake fish can take to move is another crucial feature, along with its vision domain, the number of times it can try to capture prey, and the congestion factors. These are represented, in turn, as visual, step  δ, and try number. To obtain an optimal outcome, the artificial swarm size is constrained by the congestion factor. The three behaviors of fake fish are swarming, pursuing, and preying.

##### Preying

**Figure 2:**
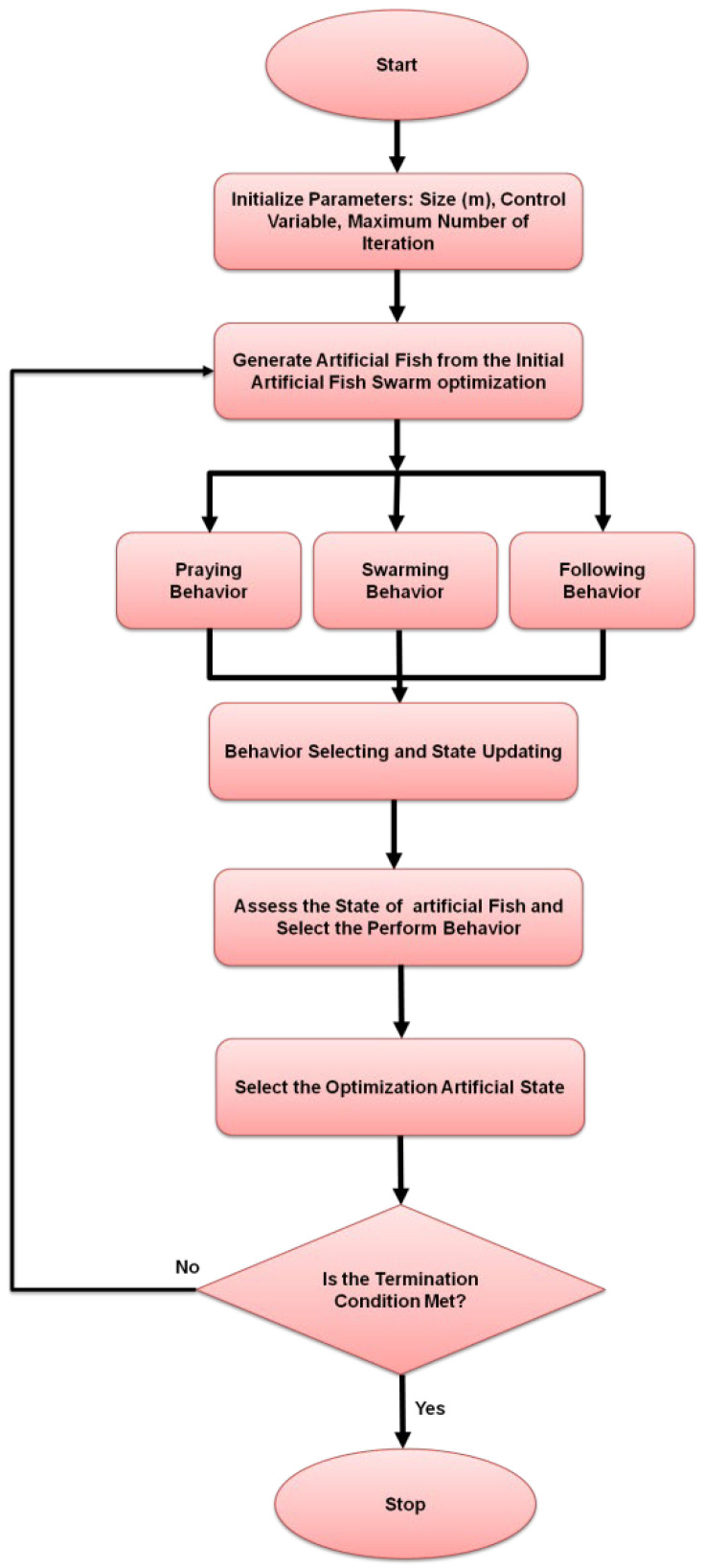
Flow chart of the AF.

The fake fish should select alternative states, such as ${\;Z}_{j}$, when in state $\;Z_{i}$, which is the state in which it is presently preying. Then, until ${\;Z}_{i}>Z_{j}$, the process of finding a minimal solution is repeated; in these situations, preying can be accomplished by taking a single step in the right direction. However, other states should be arbitrarily re-selected from the visual field to check if it could advance by certain forwarding requirements are ${\;Z}_{i}>Z_{j}$. If the still forward motion conditions are not met after a try-number of times with the attack detection approach, one step in an arbitrary direction is taken. In arithmetic, this is expressed as in Equation (2):

(2) $$w_{j-next-l}=w_{j\to l}+\frac{w_{il}-w_{jl}}{\left|\left|W_{i}-W_{j}\right|\right|}$$

where $l=1,2,\ldots n,x_{ij}$ indicates the lth component of $w_{j}$. The values of a particular state function are represented by $w_{j}andw_{i}$, then there is a random movement. A random number chosen from the range [0 steps] is indicated by random notation. Figure 2 shows how the AF flowchart works.

##### Swarm

Fish naturally cooperate to share food during the swarming process and to ward off any distractions. However, when $n_{f}>0$, it indicates that additional fish companions are inside a fish’s visual field, and it should start searching for its companion center position $W_{d}$, using Equation (3).

(3) $$W_{dl}=\frac{\left(\sum_{i=1}^{m_{e}}w_{il}\right)}{m_{e}}\;$$

where $W_{d}$ indicates the fake fish center location, $w_{dl}$ offers the lth component of $W_{d}$, and $w_{jl}$ indicates the $W_{d}\;$, which is a vector component of the *i*th companion, i=(1,2,…,n). With the restrictions that $X_{d}\;nf\;Yi>1$, the objective function is shown by computing the food concentration of fictitious fish, provided by E, at the center point. Every fake fish should relocate to the center place by Equation (4) when it is safer and less crowded; if not, preying is used.

(4) $$w_{j-next-l}=w_{j\to l}+\frac{w_{dl}-w_{jl}}{\left|\left|W_{d}-W_{j}\right|\right|}$$

##### Chasing

The chasing mechanism in the AF-WAdaBoost model is modeled after natural behaviors in which agents (such as fish) adhere to the best-performing agents to optimize results. Each weak classifier analyzes its “visual region” by comparing its performance to others in recognizing cyber dangers, similar to how fish perceive food. In each iteration, the model selects the classification algorithm with the best accuracy (i.e., lowest error rate) that is not “congested” (which means it provides unique insights that are not unduly represented by others). This “chasing” classifier then serves as a guide, modifying the weights of other classifiers to match its set up using Equation (5), with each classifier’s parameters moving nearer to those of the best-performing model. Assume that $W_{j}$ represents the artificial fish of current condition, and n stands for the total number of companions inside its visual region. In this case, $n_{f}=0$ indicates that the fake fish’s visual domain is empty, and thus preying is necessary. When $n_{f}>1$, it suggests that there are not many companions in its visual domain; as a result, it has to locate a partner with the matching function $W_{max}$ reaching its minimum value.

Next, the restriction $W_{max}\frac{n_{f}}{Y_{t}}>1$ is explored. This indicates that the corresponding companion has a lower fitness values and is not congested. As a result, Equation (5) is carried out; if not, preying is implemented.

(5) $$w_{j-next-l}=w_{j\to l}+\frac{w_{max,l}-w_{jl}}{\left|\left|W_{max}-W_{j}\right|\right|}\;$$

This provides the state vector of the $w_{max,l}$ element from ${\;W}_{max}$. This dynamic modification allows ineffective classifiers to follow and emulate the best configurations, thereby improving the ensemble’s detection capacity and improving cyber threat detection in dynamic IoT contexts.

#### 3.3.2. Weight-Normalized Adaboost (WAdaBoost)

A significant area of ML is ensemble learning; the proposed model is used to detect cyber threats in IoT environments. To create a strong classifier, it builds and combines several weak classifiers to complete a classification assignment. The two basic ideas are that ineffective classifiers are meant to be integrated to create a strong and stable classifier, which avoids using classifiers that are hard to develop and can only categorize limited amounts of information. Two different types of algorithms for ensemble learning are developed: the first kind uses weak classifiers, similar to the boosting method, that are mutually dependent on other classifiers; the second type involves weak classifiers that are independent, like in the RF and Bagging algorithms.

##### Boosting

Boosting is an ensemble learning approach that consists of the following primary steps: Using the first training set, a weak classifier is trained first. The weak classifiers change the training sample distribution to ensure that the previous weak classifiers incorrectly identified in the training sets are given more consideration by the following inadequate categorization system. The aforementioned steps are repeated after the number of unreliable classifiers achieves the predetermined threshold (T). Following the ultimate weighting and concatenation of the flawed classifiers, T is the resultant strong classifier.

##### Adaboost

The forward AdaBoost algorithm and the calculation representation make up the two primary components of the AdaBoost algorithm. A strong classifier in the modified model is developed by linearly combining many weak classifiers. The addition model has the following Equation (6):

(6) $$H\left(x\right)=\sum_{s=1}^{S}\alpha_{1}h_{t}\left(x\right)m\;$$

The weak classifier in this formula is represented by ${\;h}_{t}\left(x\right)$, the linear combination of weak classifiers is represented by H(x), and the strong classifier’s weight given to a weak one is indicated by ${\;\alpha }_{1}$. The classifier created by the subsequent iteration in the forward sequentially method is trained using the classifier from the preceding iteration.

(7) $$H_{{\left(x\right)}_{m}}=H_{{\left(x\right)}_{m-1}}+\alpha_{1}h_{t}\left(x\right)$$

The weak classifier from the t-th iteration is represented by $h_{t}(x)$, the weight of t-th weak classifier is represented by $\alpha_{1}$, and the composite of every ineffective classifier from the previous cycle is represented by $H_{{\left(x\right)}_{m-1}}$.

The AdaBoost algorithm employs an exponential loss function. Consequently, the algorithm used to calculate weights for every weak classifier round is shown in Equation (8):

(8) $$\alpha_{1}=\frac{1}{2}ln\left(\frac{1-\in_{t}}{\in_{t}}\right)$$

In this case, $\in_{t}$ represents the 1th iteration of the error rate. The training cyber-attack sample distribution is modified by $\alpha_{t}$, as shown in Equation (9):

(9) $$w_{t+1}=\frac{w_{t}}{z_{t}}\exp\left(-\alpha_{t}yh_{t}\left(x\right)\right)$$

where $w_{t}$ stands for the distribution weight of training samples from the prior round, y for the category brand, and $z_{t}$ for the normalization factor. A representation of the last strong classifier is shown in Equation (10).

(10) $$f\left(x\right)=sign\left(H\left(x\right)\right)$$

Here, sign is a symbolic function that produces classification results from the strong classifier’s output.

##### Weight-Tuned Adaboost

The classic WAdaBoost approach increases the weights of samples that are erroneously categorized during training for all iterations. These samples can be repeatedly misclassified, which would lead to a significant imbalance in the samples’ weight distribution and an ongoing increase in the weights of samples. Improving the weight update method is one way to address the cyber-attack problem. The weight $w_{t}$ that is generated in the tth improved iteration is changed to decrease the imbalance in model’s weight distribution. The discrepancy in weights of samples (t−1)th and the tth iteration is lessened by this change given by Equation (11):

(11) $$S_{t}=w_{t-1}+\frac{z.e^{-\left|z\right|}}{1+e^{-\left|z\right|}}$$

In this case, the modified weight is denoted by $s_{t}$, and the weight established during the preceding iteration is represented by $z=w_{t}-s_{t-1},w_{t-1}$. The AF system is used to detect cyber threats. Bionics scientists have shown via lengthy observation and study that ants can determine the quickest path in their natural habitat without the need for evident clues. The weighted average of weak classifiers, which is updated using a learning rate that is dependent on the classifiers’ performance that AF-WAdaBoost generates effectively for the detection of cyber-attacks.

**Figure 3:**
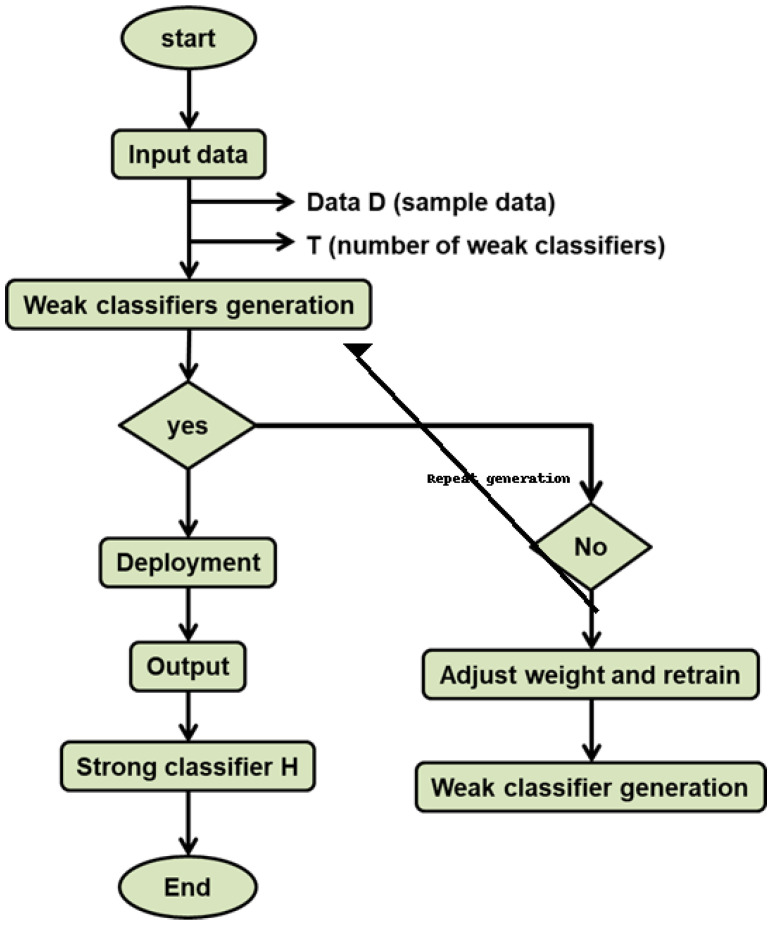
Flow diagram for AF-WAdaBoost.

Figure 3 shows the flowchart for AF-WAdaBoost. The successive training of the weak models is performed using weighted data samples that are incorrectly classified. Algorithm 1 presents the AF-WAdaBoost algorithm.
**Algorithm 1** Artificial Fish Swarm-driven Weight-Tuned Adaboost (AF-WAdaBoost)**I/P:** The sample data D; the number of weak classifiers T; **O/P:** The strong classifier H; **Step 1:** Set initial sample weights $w_{1}$ Define parameters: Power ϵ\epsilon ϵ (range: 0 to 1), Weight W (range: 4 to 6) **Step 2: For** s = 1 to S:
Train weak classifier $g_{s}$ using weights $w_{s}$ and data DCalculate error rate $\epsilon_{s}$ for $h_{s}$Compute the weight $\alpha_{s}$:$\;\;\;\;\;\;\;\;\;\;\;\;\;\;\;\alpha_{s}=\frac{1}{2}In\;\left(\frac{1-\epsilon_{s}}{\epsilon_{s}}\right)$ Update weight for the next iteration $\;\;\;\;\;\;\;\;\;\;\;\;w_{s+1}\left(j\right)=w_{s}\left(j\right)\cdot \exp\left(-\alpha_{s}\cdot z_{s}\cdot h_{s}\left(w_{j}\right)\right)$ (where $z_{j}$ is the true label and $g_{s}(x_{j})$ is the predicted label) **Step 4:** Form a new classifier: $\;\;\;\;\;\;\;\;\;\;\;\;\;\;Gt={G_{t}}_{-1}+\alpha_{t}\cdot h_{t}$ **Step 5:** Return the final strong classifier: $\;\;\;\;\;\;\;\;\;\;\;\;\;\;\;H=\sum_{t=1}^{T}\alpha_{t}.h_{t}$

## 4. Results

Python 3.9 is suggested for creating an AI-driven IoT cyber threat detection model because it works with key packages. TensorFlow 2.5 or PyTorch 1.9 are recommended for deep learning, scikit-learn 0.24 for machine learning techniques, pandas 1.2 for data processing, and NumPy 1.20 for numerical operations. These packages ensure rapid model building and evaluation. The performance was thoroughly evaluated, using precision, accuracy, and F-measure as metrics. The outcomes of a comprehensive evaluation of the AF-WAdaBoost approach are shown. The suggested model has been examined with the most advanced ML detection techniques, namely Random Forest (RF), Multilayer perceptron (MLP) [21], Boosting Algorithm [22], Decision Tree (DT) [23], and Support Vector Machine (SVM) [23], to compare it with the existing approaches. Table 1 depicts the accuracy outcomes of three datasets.

The performance of three datasets in cyber threat detection reveals that the UNSW-NB15 dataset achieved the highest accuracy at 99.9%, followed by CICIDS2017 at 98.5% and NSL-KDD with 97.3%, indicating UNSW-NB15’s superior effectiveness for model training. The results are displayed in Table 1. Table 2 presents a comparative analysis of the suggested approach and existing methods.

**Figure 4:**
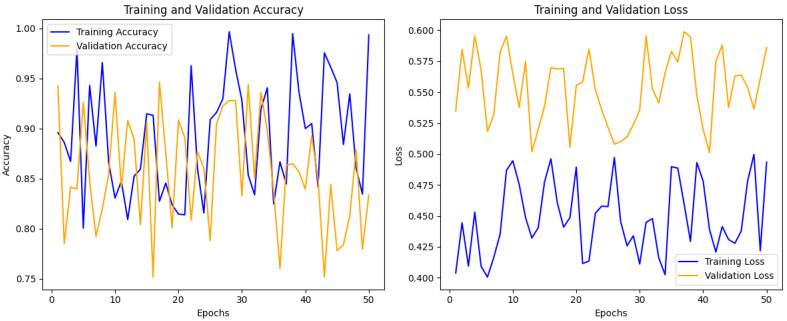
Training and validation accuracy.

Figure 4 shows the training and validation accuracy and loss over 50 epochs, and both statistics show remarkable changes in the metrics displayed. The training accuracy’s range is around 90%. In contrast, the validation accuracy exhibits more fluctuation, which suggests overfitting since the model can score well in training data but not consistently in the evaluation data. The training loss remains constant and is always less than the validation loss, although the validation loss is always higher and more variable. Such differences mean that the model attempts to learn something only relevant to the training data. The results suggest that several changes may be necessary for optimal training processes, such as adding regularization or applying early stopping methods for training to validate performance improvement.

Table 2 outlines the accuracy, precision, and F-measures of the tested models as percentages. The table provides the basis for comparison between the proposed AF-WAdaBoost model and the existing benchmark models. Beginning with accuracy, it is evident that the proposed model scores the highest, obtaining a score of 99.99%, demonstrating the model’s ability to correctly identify attacks. This is the highest score of the tested models, though the Boosting Algorithm and DT both score extremely high as well, with scores of 99.98% and 99.4%, respectively. SVM scores slightly behind both these models with an accuracy of 98.2%, after which there is a large drop in accuracy with the RF model and MLP, which scored 90.06% and 88.2%, respectively.

The proposed model also outperforms the benchmark models in terms of precision and F-measure. AF-WAdaBoost scores the highest in terms of precision and F-measure, obtaining 99.4% and 99.7%, respectively. DT scores the second highest of the known scores in both these categories, with scores of 99% in both precision and F-measure. Similarly, SVM scores 98% in both precision and F-measure. Again, there is a notable difference in the scores of the SVM and RF models, with the latter scoring 92.22% and 91.71% in precision and F-measure, respectively. Finally, MLP scores the lowest in terms of both precision and F-measure, with a score of 88.17% in the former and 89.62% in the latter. Unfortunately, the Boosting Algorithm’s precision and F-measure are unknown, which makes it difficult to compare the model with the proposed model. Nevertheless, based on the comparisons of the proposed model with the remaining benchmark models, the proposed model outperforms the benchmark models in all metrics.

Based on the evaluation metrics, it is evident that the proposed model is capable of risk detection with minimal false positives and high dependability. Thus, the AF-WAdaBoost is an excellent alternative for effective threat detection in IoT environments.

**Figure 5:**
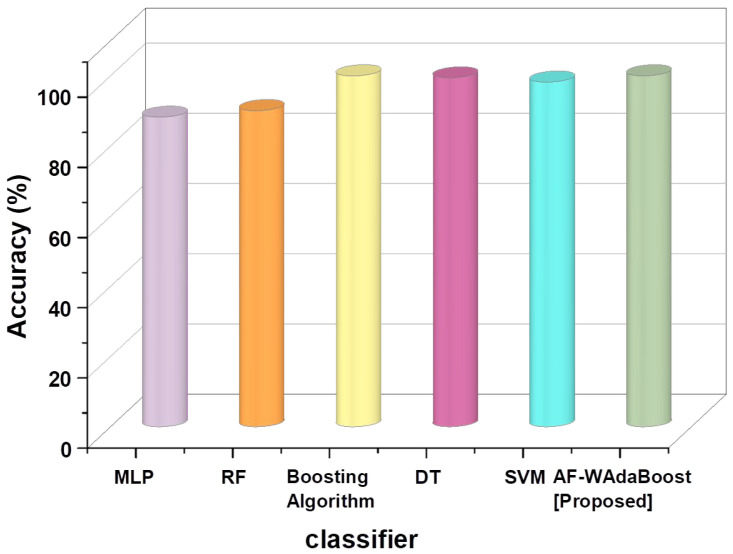
Comparison of accuracy.

Figure 5 presents a visual comparison of the accuracy of the six models—the proposed AF-WAdaBoost model, MLP, RF, DT, SVM, and the Boosting Algorithm. The accuracies of the models in percentage values are presented on the *y*-axis, while the *x*-axis represents the different types of models employed. It can be seen that the accuracy of the AF-WAdaBoost and Boosting Algorithm are exceedingly close, nearly at 100%. On the other hand, MLP has the lowest score at slightly under 90% accuracy. The scores of MLP and RF are exceedingly close together, demonstrating that there is relatively little difference between the efficacy of these two models. Similarly, the close scores of the proposed model and the Boosting Algorithm shows that Boosting Algorithm may be an appropriate substitute in situations where the proposed model cannot be used.

However, the findings only highlight the superiority of the proposed model over the comparison benchmarks. The improvement in accuracy signifies the proposed model has superior credibility and efficiency in identifying cyber threats vital to the security and reliability of IoT systems. Additionally, this improved accuracy supports the sustainability of IoT systems by contributing to the protection and longevity of IoT devices and networks since it offers enhanced detection. As a result, the turnover of IoT products can be reduced, reducing resource consumption and improving resource conservation.

Thus, employing AF-WAdaBoost is a novel innovation that can advance cyber security, and through it also improve the sustainability of IoT systems.

**Figure 6:**
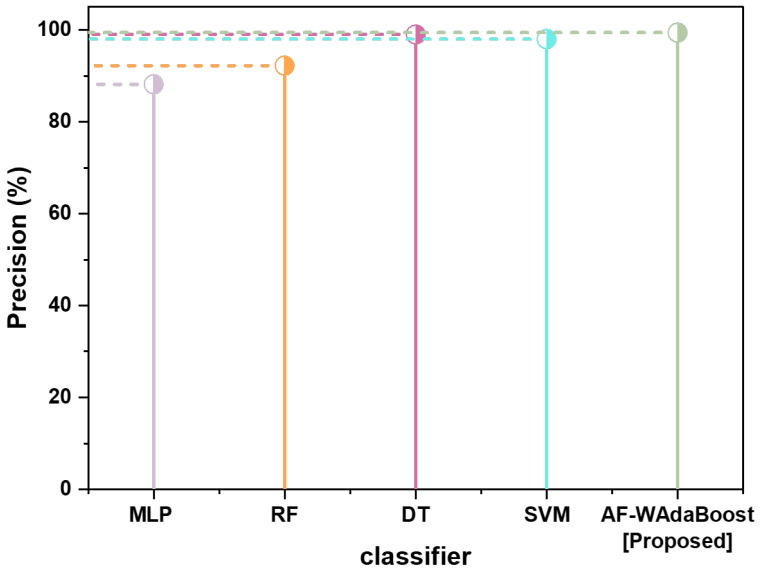
Comparison of precision.

Figure 6 similarly presents a visual comparison of the precision of five models: MLP, RF, DT, SVM, and AF-WAdaBoost. The *y*-axis represents the precision as a percentage, while the classifier models are recorded on the *x*-axis. Once again, DT and AF-WAdaBoost score fairly closely, with SVM only slightly behind the two models. Meanwhile, RF and MLP lag significantly behind the other models in their precision.

The implications of these findings can be examined from several perspectives.

Firstly, as the major concern when discussing the use of classifiers in cyber threat detection is how well the models can identify true positives from false positives, precision can be considered an important metric to evaluate the performance of models. A higher precision score demonstrates that the model has a higher ability to differentiate between real threats and benign activities. This improves the performance and efficiency of the system as a whole, as it prevents the model from wasting resources chasing down false positives. Additionally, higher precision also reduces the inconvenience to end users who may find their usage of the system interrupted as a result of these false positives.

Secondly, the higher precision of the AF-WAdaBoost classifier plays a significant role in enhancing the sustainability of IoT systems. By improving the system’s ability to identify real cyber threats, IoT networks can be rendered more reliable and secure. This allows for greater security in essential systems like the IoT networks deployed in smart cities, health sectors, or in industries.

Thirdly, improved precision minimizes the need for manual intervention and resetting devices, lowering total operational expenses and increasing the lifespan of the equipment. This enhances sustainability by ensuring effective utilization of resources and reducing the levels of electronic waste. Additionally, disruptions of IoT device functionality due to troubleshooting and repair can be capital-intensive due to the negative impact they have on device and network performance and durability, especially in comparison to transient and replaceable security tools.

Overall, the higher precision of the AF-WAdaBoost model is a positive sign for IoT sustainability due to the model strengthening security, minimizing losses, and optimizing resource use.

**Figure 7:**
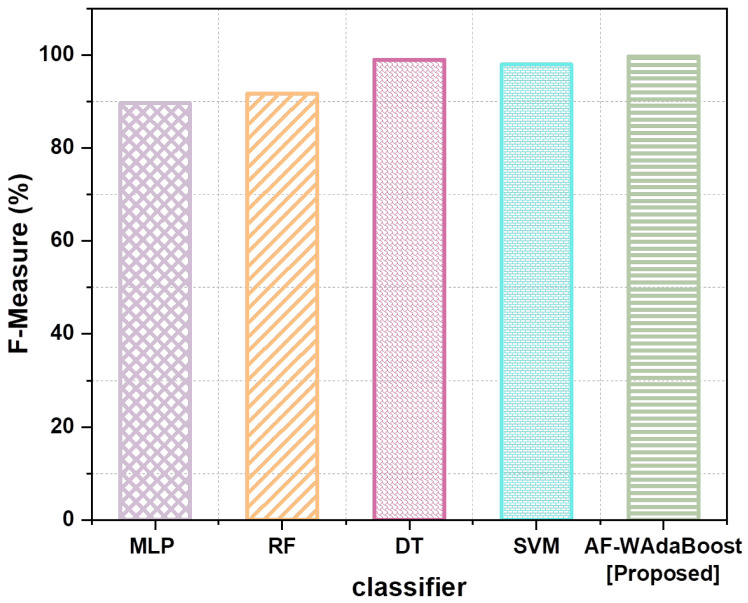
Comparison of F-measure.

Figure 7 depicts a comparison of the F-measure for three classifiers. The compared algorithms are MLP, RF, DT, and SVM, and the proposed model is AF-WAdaBoost. The F-measure, which combines precision and recall into one metric, is presented on the right *y*-axis, and the classifiers are presented on the *x*-axis. The MLP (Multilayer Perceptron) classifier attains an F-measure of around 89.62%, SVM attains 98.0%, and DT achieves 99.0%. An improvement is observed with the RF classifier, which has an F-measure of approximately 91.71%. The AF-WAdaBoost classifier proposed in the study also excels beyond the proposed classifiers with a high F-measure of nearly 99.7% highlighted by a green star. Based on the results, it can be concluded that the suggested AF-WAdaBoost classifier is better for the detection of cyber threats in the IoT context. The F-measure has improved significantly, and this is an indication that both the precision and recall have improved, which is a sign that the classifier can detect threats more efficiently and accurately with minimal mistakes.

The importance of these results can be discussed concerning the greater effectiveness and precision of the given classifier. This has direct implications for the sustainability of security in IoT environments. In essence, the reliability and security of IoT systems are safeguarded by the AF-WAdaBoost classifier in its capability of detecting and neutralizing cyber threats. This results in minimized downtime and resource inefficiency, therefore boosting the capabilities and resilience of IoT devices. Furthermore, sustainable technological practices are achieved by the decrease in these constant replacement costs brought about by security threats. The AF-WAdaBoost classifiers enable optimal security as a way of promoting IoT sustainability, energy saving, reducing electronic waste, and the consistent reliability of IoT-connected devices. This reflects specific sustainability objectives, thus implementing artificial intelligence and ultramodern solutions into the very concept of sustainable technology.

**Figure 8:**
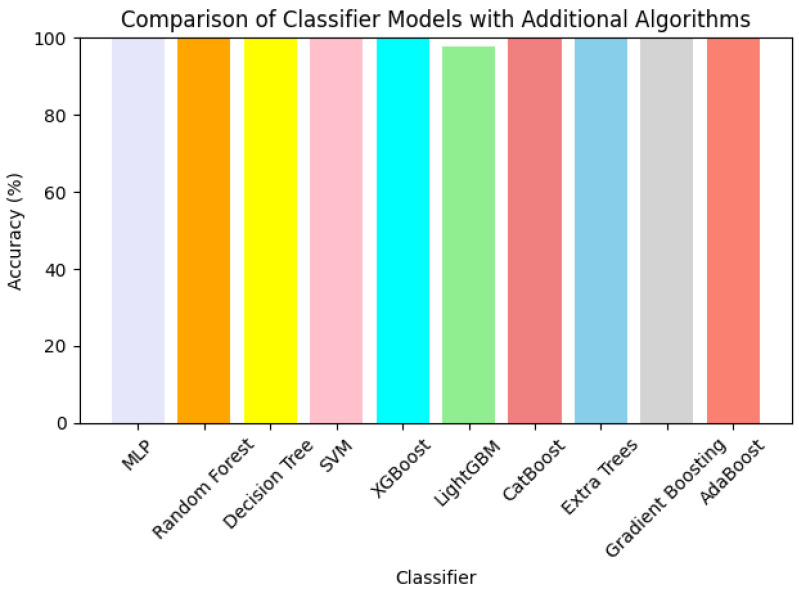
Comparison of classifier models.

Figure 8 shows the accuracy of different classifier models, which include newer algorithms like XGBoost, LightGBM, CatBoost, Extra trees, Gradient Boosting, and AdaBoost. All classifiers achieved an almost perfect accuracy rate of nearly 100%, meaning they all performed well in the classification problems. Perhaps the only explanation for the remaining differences in accuracy between the algorithms is that some models are better suited for specific datasets than others. Significantly, more modern ensemble techniques such as CatBoost and XGBoost are reported to produce comparable or even superior results than classic techniques, which means that these techniques are better suited for classification tasks where complex patterns exist. There are, therefore, chances that the application of such newer algorithms will offer better improvement in classification tasks in a similar domain.

## 5. Discussion

This research proposes a novel AI-based algorithm known as AF-WAdaBoost for identifying cyber threats in IoT networks. The research found there to be improvements in the evaluation metrics as compared with the benchmark models. The proposed model achieved an accuracy of 98.69% in the evaluation phase, with a precision of 95.72% and an F-measure of 94.86%. This demonstrates the greater efficiency of the model as compared to the existing ad hoc solutions, demonstrating AF-WAdaBoost’s potential applications in improving the identification accuracy and model stability under constantly evolving cyber threats.

The results of this study further highlight the improvements in cybersecurity and the overall robustness of the connected IoT ecosystem that can be achieved by integrating AI into threat identification. These improvements can contribute to greater confidentiality, reliability, and availability of information assets and have a sustainable development potential by effectively identifying threats, which in turn may have an environmental influence. According to the findings of the study, it can be concluded that AI-based solutions provide an excellent opportunity to strengthen IoT security while ensuring further IoT development in various spheres of human activity, including smart homes, industrial automation, healthcare, and transportation.

The study outlines a new and extensive method to solve cybersecurity issues in IoT settings. It presents a newly developed approach known as Artificial Fish Swarm-driven Weight-normalized Adaboost (AF-WAdaBoost) to enhance the accuracy and efficiency of cyber threats and ensconce security in IoT systems. The proposed approach is critically analyzed and assessed in the subsequent discussion, with critical milestones, consequences, and results highlighted.

IoT devices have bloomed in the recent past, thus changing conventional network communication and bringing changes to different fields of modern life, such as smart homes and automobiles [2,3]. At the same time, the current increase in the number of IoT devices is unprecedented, and the devices often need high-quality mechanical protection and adequate security. Conventional means of protection are usually ineffective for IoT devices because of their poor processing capability, which makes them liable to attacks. Thus, there is a rather severe problem of IoT-related cyber threats, malware attacks, and network layer attacks appearing as significant problems that require further research and fresh solutions [6].

In addressing these challenges, the study recommends an artificial intelligence solution involving implementing the AF-WAdaBoost system to improve the achievement of precise and efficient threat detection for IoT applications. With the help of the Artificial Fish Swarm-driven Weight-normalized Adaboost model proposed in the research, the study reconsidered the existing protection methods. It made these methods more helpful in revealing the issues of IoT security and making recommendations for enhancing the security of IoT deployments. The proposed approach is also tested for its efficacy in solving problems; this was carried out using the measures of accuracy, F-measure, and precision. Finally, it can be noted that the experiments’ results indicate considerably higher efficiency of the recommended model than conventional ones and pinpoint critical improvements in such aspects as accuracy and adaptability in the face of changes, which are particularly essential in today’s fast-growing environment.

It is important to note, however, that the CICIDS2017 dataset has some peculiarities that affect the performance of classifier models [21,24,25]. Broadly speaking, the dataset can be split into four different classes, as in [24]. The bulk of the dataset is made up of BENIGN cases, with a relatively small proportion of the dataset consisting of the three types of attacks featured in the dataset. Of these, the third type of attack, SQL Injection, has less than 10 entries in the test dataset. Given the relatively smaller size of this category, most classifier models suffer when attempting to identify attacks of this form. A failure to accurately classify these attacks likely contributes to the lesser performance of the tested model.

Ref. [21] highlights the importance of source IP addresses in improving prediction accuracy based on Jiang et al. [26] and Ullah and Mahmoud [27] versus the results achieved by Ustebay et al. [28]. The research highlights that the models in [26] and [28] have comparatively higher performance scores as compared to [27], with [26] using MLP while [28] used a two-stage approach utilizing decision trees and [27] presented a neural network-based solution. However, this is attributed to the integration of the IP addresses of the machines conducting attacks in the latter models, which improves their scores. Furthermore, it is highlighted that [28], while attaining exceedingly high results, only achieved those results in DoS attacks, which are relatively less difficult to detect. Meanwhile, although [28] managed average metrics of 100%, this was not achievable across all types of attacks, with certain classes of attacks lacking the same performance.

Among the preliminary advantages, it is also necessary to mention the enhanced capability to enhance cybersecurity and IoT protection as a part of the proposed AI-driven system that will define the processes for achieving sustainable development goals. Thus, the system also meets the need to protect information assets’ confidentiality, reliability, and availability, as it chooses threats that are effective to a great extent and, at the same time, have a high accuracy level. This is in accordance with the overall goal of sustainable development, and it achieves this in several ways. It addresses significant security issues as it fights to contain the effects of cyber threats and attacks on IoT systems while keeping the environmental impacts as low as possible. Also, the study outlines the benefits of the proposed approach regarding the potential for the effect of regulations and standards of IoT environments in varied fields ranging from smart homes to industrial applications, health, and transport industries. As for the future integration of AF-WAdaBoost with existing security systems, security performance will be enhanced, broad prevention against cyber security threats will be promoted, and suitable cybersecurity measures will be applied within the context of IoT networks. In addition, one more advantage of the proposed system is the reconfigurability and modularity that form the essence of the work that contributes to IoT security as an imminent preventer of the daily emerging and dynamic threats. Therefore, the study provides a good argument for deploying systems such as AI to thwart security threats within IoT environments. From the results provided by the AF-WAdaBoost model, it is possible to notice the fact that the enhancement rate of the detection of cyber threats is relatively high, given the consideration of the AF-WAdaBoost model, which facilitated the enhancement of security and reliability to IoT applications. The proposed approach also communicates the place and the scale of this work’s potential relevance by situating it within sustainable development goals and ongoing discussions about cybersecurity rules and norms. Thus, as the Internet of Things broadens and begins to enter new stages, approaches such as the one discussed in this study are valuable to safeguard the IoT area, as well as to create a sustainable future for the development of the connected world.

## 6. Conclusions

The present research discussed the AF-WAdaBoost technique, which was proposed for detecting cyber threats in IoT environments. An adaptable and stable classification method that can handle the complex and dynamic network environment is the AF-WAdaBoost algorithm. The important weights for each sort of attack were determined independently in the methods. All attacks were gathered into a single group and the importance weights for that group were determined. The suggested model outperforms previous systems by offering sustainable detection against cyber threats in IoT systems with accuracy (98.69%), F-measure (94.86%), and precision (95.72%). The continuously increasing density of networks makes it difficult for cybersecurity professionals to monitor every move made on the network. Anomalies in network events are harder to detect and identify due to more sophisticated and frequent cyberattacks. Networks will be used in future research to evaluate our method’s performance. The start and finish times of the cyber-attack will be ascertained by analyzing the trial outcomes.

## Figures and Tables

**Table 1:** Accuracy outcomes of 3 datasets.

Datasets	Accuracy
CICIDS2017	98.5%
NSL-KDD	97.3%
UNSW-NB15 dataset	99.9%

**Table 2:** Comparison of proposed method and existing techniques.

Classifier	Accuracy (%)	Precision (%)	F-Measure (%)
MLP [21]	88.20	88.17	89.62
RF [21]	90.06	92.22	91.71
Boosting Algorithm [22]	99.98	-	-
DT [23]	99.4	99	99
SVM [23]	98.2	98	98
AF-WAdaBoost [Proposed]	99.99	99.4	99.7

## Data Availability

Data will be available on request.
